# Sex differences in thermophysiological responses of elderly to low-intensity exercise during uncompensable heat strain

**DOI:** 10.1007/s00421-024-05457-8

**Published:** 2024-03-29

**Authors:** Hein A. M. Daanen, Iris Dijkstra, Emma Abbink, Iris J. de Jong, S. Tony Wolf, Coen C. W. G. Bongers, Laurens S. Hondema, Thijs M. H. Eijsvogels, Boris R. M. Kingma

**Affiliations:** 1 Department of Human Movement Sciences, Faculty of Behavioural and Movement Sciences, Amsterdam Movement Sciences, Vrije Universiteit Amsterdam, Van der Boechorststraat 7, 1081BT Amsterdam, The Netherlands; 2 Department of Kinesiology, University of Georgia, Athens, GA USA; 3 School of Sport and Exercise, HAN University of Applied Sciences, Nijmegen, The Netherlands; 4 Department of Medical Biosciences, Exercise Physiology Research Group, Radboud University Medical Center, Nijmegen, The Netherlands; 5 Public Health Service of Amsterdam (GGD), Amsterdam, The Netherlands; 6 Department Human Performance, Unit Defence, Safety and Security, TNO, Organization for Applied Sciences, Soesterberg, The Netherlands

**Keywords:** Core temperature, Environmental extremes, Exercise, Sweat rate, WBGT

## Abstract

**Purpose:**

The rising frequency of extreme heat events poses an escalating threat of heat-related illnesses and fatalities, placing an additional strain on global healthcare systems. Whether the risk of heat-related issues is sex specific, particularly among the elderly, remains uncertain.

**Methods:**

16 men and 15 women of similar age (69 ± 5 years) were exposed to an air temperature of 39.1 ± 0.3 °C and a relative humidity (RH) of 25.1 ± 1.9%, during 20 min of seated rest and at least 40 min of low-intensity (10 W) cycling exercise. RH was gradually increased by 2% every 5 min starting at minute 30. We measured sweat rate, heart rate, thermal sensation, and the rise in gastrointestinal temperature (Tgi) and skin temperature (Tsk).

**Results:**

Tgi consistently increased from minute 30 to 60, with no significant difference between females and males (0.012 ± 0.004 °C/min vs. 0.011 ± 0.005 °C/min; *p* = 0.64). Similarly, Tsk increase did not differ between females and males (0.044 ± 0.007 °C/min vs. 0.038 ± 0.011 °C/min; *p* = 0.07). Females exhibited lower sweat rates than males (0.29 ± 0.06 vs. 0.45 ± 0.14 mg/m^2^/min; *p* < 0.001) in particular at relative humidities exceeding 30%. No sex differences in heart rate and thermal sensation were observed.

**Conclusion:**

Elderly females exhibit significantly lower sweat rates than their male counterparts during low-intensity exercise at ambient temperatures of 39 °C when humidity exceeds 30%. However, both elderly males and females demonstrate a comparable rise in core temperature, skin temperature, and mean body temperature, indicating similar health-related risks associated with heat exposure.

## Introduction

A significant rise in mortality, particularly among the elderly population, has been observed during heatwaves (Robine et al. 2008; Semenza et al. 1996). Although most studies indicate that females face a heightened mortality risk during heatwaves (Ballester et al. 2023; Folkerts et al. 2022; van Steen et al. 2019), some have demonstrated higher mortality risk in males (Khatana et al. 2022; Vaidyanathan et al. 2020). Females exhibit lower sweat gland output and less evaporative heat loss than males during heavy exercise (Gagnon 2012) resulting in less effective cooling in particular in dry heat. Gagnon and Kenny (2012) imposed a relative humidity of 12% which made it easy to evaporate the produced sweat. Additionally, females show an earlier onset of body core temperature rises when dehydrated compared to males (Wickham et al. 2021). However, the lower muscle mass and smaller body size of women lead to less heat generation and, therefore, require less sweat output in high ambient temperatures leading to no inherent disadvantage (Yanovich et al. 2020). On the other hand, body surface area of females is lower than that of males, thus reducing the area over which sweat can be effectively evaporated. Consequently, the unresolved nature of sex differences in vulnerability to heat exposure persists.

Humidity ramp protocols are commonly employed to assess human vulnerability to heat (e.g., Kenney and Zeman 2002; Ravanelli et al. 2018). In this protocol, humidity is incrementally increased to determine the environmental conditions (temperature and humidity) at which the core body temperature exhibits a significant rise. This shift is interpreted as the transition from compensable heat strain to uncompensable heat strain (Wolf et al. 2021a; Cottle et al. 2023; Cottle et al. 2022). The threshold from compensable to uncompensable heat strain is higher in unacclimatized females than males in wet humid climates exercising at 30% *V*O_2_max, but not in hot dry climates (Kenney and Zeman 2002; Wolf et al. 2022). The threshold for males was about 31 °C WBGT ((indoor) wet-bulb globe temperature) and close to 33 °C for females in a wet humid climate (Kenney and Zeman 2002). The reason for this difference is likely that the reported metabolism of females is much lower (about 215 W) than that of males (about 400 W) at 30% *V*O_2_max; and, therefore, also require a lower amount of sweat to evaporate to compensate for the heat production. In hot dry climates, the limit of evaporative heat loss is related to the amount of sweat that is produced, therefore, females have a relative disadvantage in hot dry conditions because on average they cannot sweat as much as males. While the thresholds from compensable to uncompensable heat strain are described, also for elderly recently (Wolf et al. 2023), the thermophysiological responses to heat in elderly males and females during uncompensable heat strain have not been investigated.

Therefore, we conducted a study to investigate differences in thermophysiological responses to low-intensity exercise in the heat between healthy elderly (60 years and older) males and females. Considering that elderly people have a considerable earlier transition from compensable to uncompensable heat strain that young people in increasing heat (Wolf et al. 2023), we expected that the elderly would be exposed to uncompensable heat strain with ambient temperatures of 39 °C and relative humidities increasing stepwise from 25% onwards. Non-weight bearing exercise (cycling) was chosen to avoid differences in metabolic rate due to differences in body weight (Yanovich et al. 2020) and enable a fair comparison of heat loss mechanisms between sexes when exposed to heat. We hypothesized that no sex differences would exist in core, skin and mean body temperature during uncompensabe heat strain, and no sex differences in sweat rate for low humidities, but higher sweat rates in males for high humidities in line with observations from Kenney and Zeman (2002) in young adults.

## Materials and methods

### Participants

Participants in this study were healthy unacclimatized elderly above the age of 60 years who were recruited through community centers and sports centers in Amsterdam. A medical assessment took place before the start of the experiments. Participants were excluded if they (i) suffered from lung or heart disease; (ii) used medication that could influence their thermoregulatory responses to heat (e.g. beta blocking agents); (iii) had spent over a week in a hot environment in the last two months; (iv) had experienced exertional heat stroke in the past; and (v) did not meet the requirements tested during the medical assessment (e.g. abnormal results in electrocardiogram).

After assessing the participants for inclusion, 31 participants (16 men and 15 women) were included in the study. Men and women were comparable in age (*p* = 0.561), surface area-to-mass ratio (AD/kg) (*p* = 0.299), and BMI (*p* = 0.364). Females were extremely likely to be above the age of menopause (Rödström et al. 2005). Table 1 shows an overview of the overall and sex-disaggregated subject characteristics. All experimental procedures received ethics approval from a Medical Ethical Review Committee (NL69479.078.19). After the participants were informed about the content of the study and the experiments, written informed consent was obtained.

**Table 1:** Overall and sex-disaggregated means and standard deviations of subject characteristics

	All	Women	Men
*n*	31	15	16
Age (year)	69 ± 5	70 ± 5	68 ± 5
Height (m)	1.72 ± 0.11	1.65 ± 0.07	1.79 ± 0.09*
Weight (kg)	71.0 ± 12.9	64.1 ± 12.2	77.5 ± 10.0*
BMI (kg/m^2^)	23.8 ± 2.6	23.3 ± 3.3	24.2 ± 1.8
AD (m^2^)	1.84 ± 0.22	1.71 ± 0.18	1.96 ± 0.17*
AD/kg (m^2^/kg)	0.027 ± 0.0046	0.028 ± 0.0041	0.026 ± 0.0050

Data were analyzed using unpaired samples *t* tests

*BMI* body mass index (kg/m^2^), *AD* dubois surface area (m^2^)

*P <  0.05 compared with women

### Procedures

Participants were asked to visit the test location two times. The first visit consisted of medical assessment and a submaximal exercise test on an ergometer to assess physical fitness using the YMCA test (Golding et al. 1989).

During the second visit, the participants were exposed to 39.1 ± 0.3 °C and 25.1 ± 1.9% relative humidity (RH) in a climate chamber (b-Cat B.V., Tiel, The Netherlands). After 20 min of rest, the participants started cycling with a load of 10 W at a cadence of 70–90 rpm (Lode Excalibur, Groningen, The Netherlands). 30 Minutes after entering the climate chamber, RH was increased by 2% (about 1 mmHg water pressure) every five minutes. The maximal duration of the trial was 135 min. However, the experiment was terminated earlier if gastro-intestinal temperature (Tgi) exceeded 39.5 °C or if Tgi rose by > 0.1 °C every 5 min for 15 consecutive minutes with minimal duration of the experiment of 60 min. The values for temperature and humidity were chosen based on the observation that the inflection point in Tgi occurred for 5% of the elderly at relative humidities of about 25% and averaging about 35% in ambient temperatures of about 39 °C (Wolf et al. 2023).

The participants were asked to not consume alcohol and caffeine and to not participate in intensive exercise 12 h prior to the experiments. The participants arrived at the research facility one hour prior to the start of each experiment. At arrival, they were requested to provide a urine sample to ensure euhydration, defined as a urine-specific gravity < 1.025 g/L (USG; PAL-S, Atago, Bellevue, WA) (Wolf et al. 2021b). After euhydration was ensured, participants were asked to ingest an intestinal temperature capsule which measured gastrointestinal temperature (Tgi). Afterwards, the participants’ semi-nude weight and the weight of the clothes that the participant was going to wear during the experiment were determined. The clothes consisted of a T-shirt, shorts, socks, and shoes. Directly after the end of the experiment, the semi-nude body weight and the weight of the clothes were measured again.

### Measurements

RH and ambient temperature (Tamb) were measured using a data logger that was located in the middle of the climate chamber. WBGT values were calculated using the software tool available at http://www.climatechip.org/excel-wbgt-calculator. Tgi was measured with a validated intestinal temperature capsule (Bongers et al. 2018) which was ingested by the participants one hour prior to the start of the experiment (BodyCap, Caen, France). Skin temperature (Tsk) was measured using iButtons (DS1922L, Maxim Integrated Products Inc, Sunnyvale, CA, USA) (van Marken Lichtenbelt et al. 2006). The iButtons were applied to 8 different parts of the body before the start of the experiments. The weighing factors to determine Tsk are 0.05 for hand, 0.07 for forehead and left and right arm, 0.175 for scapula and chest, 0.19 for thigh and 0.20 for calf according to the ISO 9886 standard (ISO_9886 2004).

Oxygen uptake (COSMED Quark CPET, Italy) was obtained twice during the cycling period from minutes 30–35 and minutes 55–60 and values were averaged. Metabolic heat production (M; W/m^2^) was calculated from oxygen consumption (*V*O_2_; L/min/kg) and respiratory exchange ratio (RER):

${\text{M}} = VO_2 \cdot \frac{{\left[ {\left( {\left( {\frac{RER - 0.7}{{0.3}}} \right)*21.13 + \left( {\frac{1.0 - RER}{{0.3}}} \right)*19.62} \right)} \right]}}{60}*1000* A_D^{ - 1}$, where A_D_ is Dubois surface area (m^2^).

Mean body temperature Tbody was calculated with the following formula:

$${\text{Tbody }} = \, 0.{8 }*{\text{ Tgi }} + \, 0.{2 }*{\text{ Tsk}}$$

The clothed body weight and clothing weight (including a towel) were determined separately prior to and after exercise on a scale accurate to 5 g (Platform scale, SATEX 34 SA-1 250, Weegtechniek Holland BV, Zeewolde, The Netherlands). Clothing weight gain was subtracted from body weight loss to assess whole body evaporative sweat loss. A towel was used to absorb sweat dripping of the body during the experiments and participants were not toweled off before weighing. Participants were not allowed to drink anything between the first and final body mass measurement. The whole body evaporated sweat rate (WBSR) was determined by dividing whole body evaporative sweat loss by the surface area, as calculated by the formula of Dubois (Du Bois and Du Bois 1916), and the total duration of the experiment.

Local evaporative sweat rate (LSR) was measured using a ventilated capsule system on medial side of the middle of the right forearm. The capsule was fixed to the skin using medically certified collodion glue (Collodion USP, Mavidon, Riviera Beach, FL, USA). Dry nitrogen gas was passed through the capsule at a known flow rate, with the help of a flow meter (Omega Engineering, Stanford, CT, USA). The flow rate determines how much sweat is being evaporated under the capsule per time-unit, the higher the flow rate the faster the evaporation. The flow of the influent dry nitrogen gas was maintained at a constant rate of 102.1 mL/min. Approximately 1 m downstream of the capsule, the temperature and RH of effluent air are measured (HygroVUE10, Campbell Scientific, Logan, UT, USA). Mean LSR was calculated over the total duration of the experiment.

Thermal sensation (TS) and thermal comfort (TC) were monitored every 5 min. TS was measured on a 9-point scale ranging from − 4 very cold to + 4 very hot. TC was measured on a 5-point scale ranging from 0 comfortable to 4 extremely uncomfortable (ISO_10551 1995).

### Statistical analysis

The data were analyzed in Statistica (TIBCO_Software_Inc 2020). The differences between males and females for anthropometry, fitness level, experiment duration, Tgi, Tsk, Tbody, sweat rate and subjective ratings were analyzed using an unpaired samples t-test when the data were normally distributed according to the Kolmogorov–Smirnov test and a Mann–Whitney test when not-normally distributed. Significance was set at *p* < 0.05.

Pearson correlation coefficients were calculated to determine the relation between WBSR and LSR. For this purpose, the LSR values were cumulative for each individual over the entire measurement period.

## Results

**Fig. 1:**
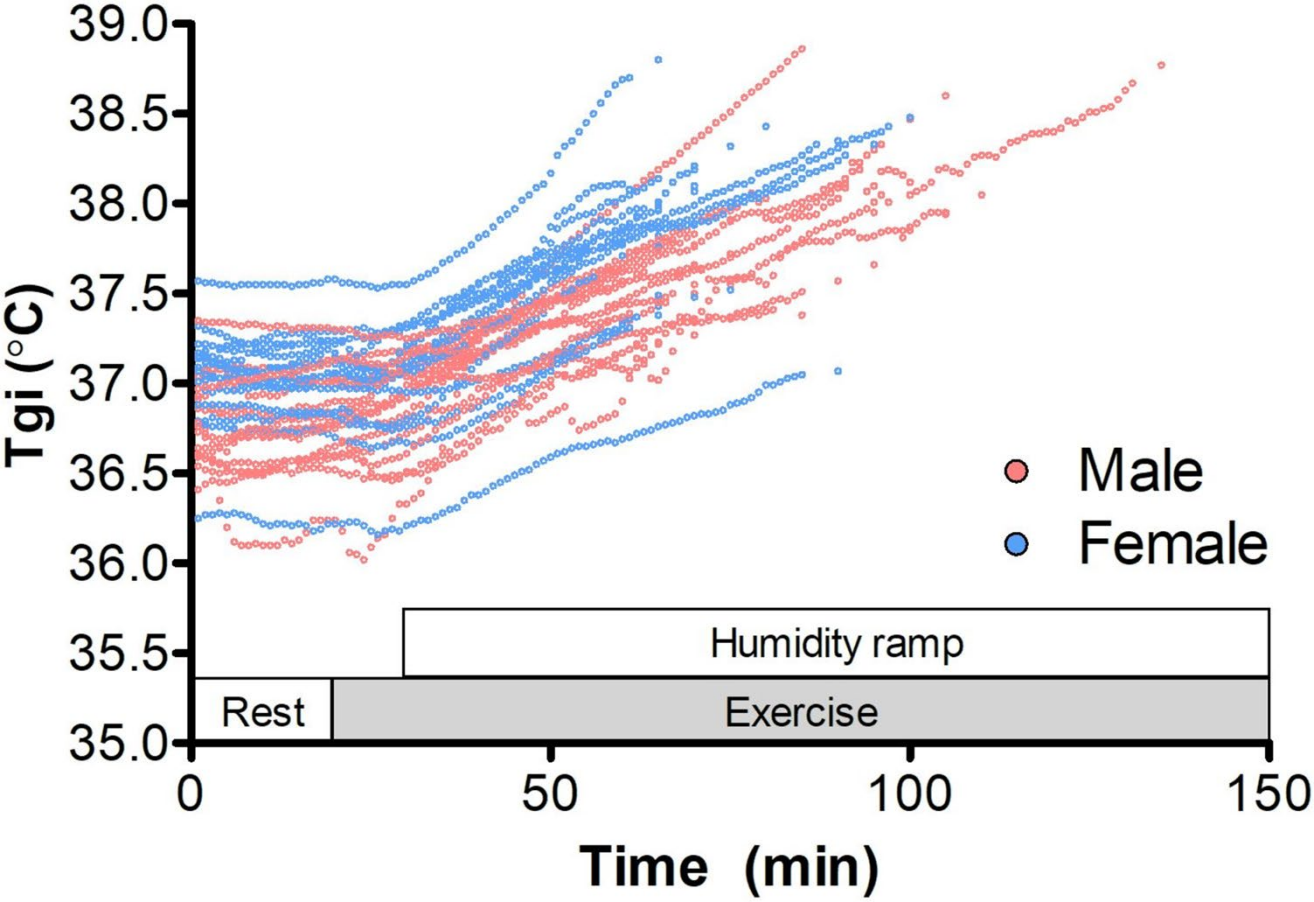
Gastrointestinal temperatures for each participant. Exercise started after 20 min in the climatic chamber and humidity was increased stepwise after 30 min. Males are in red, female in blue. All participants were at least 60 min in the climatic chamber

The estimated VO_2_max using the YMCA test was approximately 35 ml/kg/min for females and 36 ml/kg/min for males (*p* = 0.69). The experiment lasted 74.5 ± 12.7 min for females and 86.9 ± 18.4 min for males on average (*p* = 0.04). Each participant was in the climatic chamber for at least 60 min. No participant reached Tgi values exceeding 39.5 °C so the termination was based on a Tgi rise of > 0.1 °C every 5 min for 15 consecutive minutes for all participants. Heart rate data were missing due to technical failure for one male and one female participant, metabolism for one female participant and Tgi for one male participant; all other data were acquired successfully. Tgi of all subjects increased after 20–30 min in the climatic chamber (Fig. 1). The individual data were shown to illustrate that the participants were experiencing uncompensable heat strain.

**Fig. 2:**
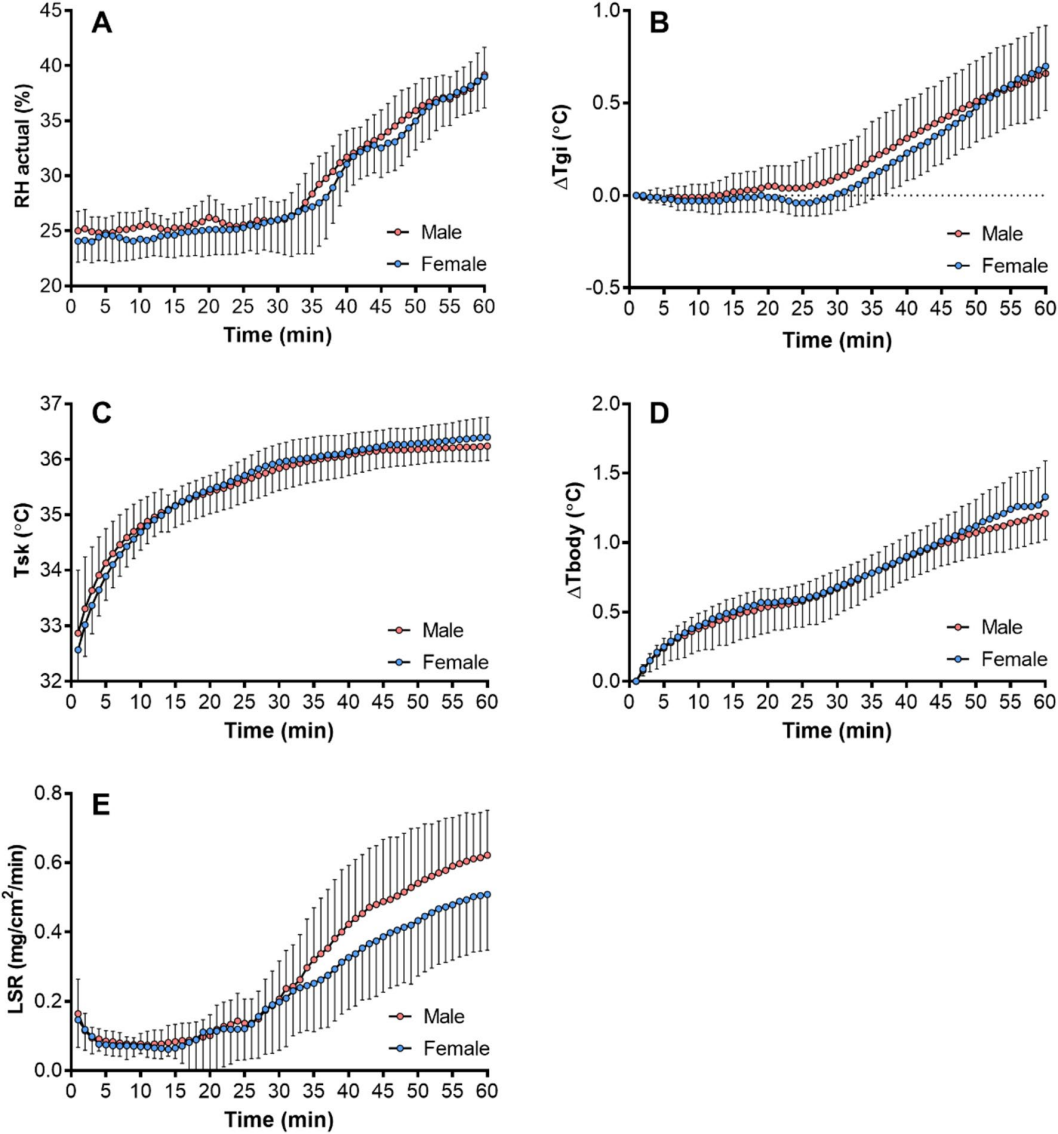
Relative humidity (RH) in % (panel A), increase in gastrointestinal temperature (GI) in °C (panel B), mean skin temperature (Tsk) in °C (panel C), increase in mean body temperature (Tbody) in °C (panel D), local sweat rate (LSR) in mg/cm^2^/min (panel E) versus time averaged over the male and female participants. Vertical bars indicate the 95% confidence interval. *n* = 15 for females and *n* = 16 for males for panels A, C, and E; *n* = 15 for females and *n* = 15 for males for panels B and D

Figure 2 shows (the increase in) Tgi, Tsk, Tbody, and LSR against time for males and females. The first panel shows relative humidity as a reference. No sex differences were observed in increase in Tgi, Tsk, Tbody. However, LSR increases considerable more for males than females after minute 32, in particular for relative humidities exceeding 30%.

Table 2 shows the main results for males and females regarding metabolism, heart rate, temperatures and thermal sensation/thermal comfort for the 60 min period. Metabolism (in W/m^2^) did not differ between males and females (*p* = 0.10), and since the external power was fixed at 10 W, also absolute heat production did not differ between sexes (*p* = 0.75). Tgi was not significantly different between females and males (0.012 ± 0.004 °C/min vs. 0.011 ± 0.005 °C/min; *p* = 0.64). The increase in Tsk also did not differ between females and males (0.044 ± 0.007 °C/min vs. 0.038 ± 0.011 °C/min; *p* = 0.07). Females exhibited lower whole body sweat rates than males (0.29 ± 0.06 vs. 0.45 ± 0.14 mg/m^2^/min; *p* < 0.001) in particular at relative humidities exceeding 30%. WBSR and LSR were well correlated (*r* = 0.54, *P* < 0.002). Thermal sensation and thermal comfort did not differ between sexes (*p* = 0.30 for both).

**Table 2:** Mean values and Standard Deviation (SD) and Hedges’ g of predicted *V*O_2_max, metabolism (in W and W/m^2^), gross efficiency, whole body sweat rate (WBSR), local sweat rate (LSR), resting heart rate (HRrest), heart rate after 60 min in the climatic chamber (HR60), resting mean skin temperature (Tsk-rest), mean skin temperature after 60 min in the climatic chamber (Tsk60), increase in mean skin temperature (Tsk increase), resting gastrointestinal temperature (Tgi-rest), gastrointestinal temperature after 60 min in the climatic chamber (Tgi60), increase in Tgi (Tgi increase), resting mean body temperature (Tbody-rest), mean body temperature after 60 min in the climatic chamber (Tb60), and increase in mean body temperature (Tbody increase), thermal sensation frp, 0–60 min in the climatic chamber (TS 0–60) and thermal comfort from 0 to 60 min in the climatic chamber (TC 0–60). The right column indicates the significance level of the t-test or the non-parametric Mann–Whitney test (indicated with #). Rest values are averages over the first 20 min. *n* = 15 for females and *n* = 16 for males. For heart rate *n* = 14 for females and *n* = 15 for males. For metabolism *n* = 14 for females and *n* = 16 for males. For predicted *V*O_2_max *n* = 9 for females and *n* = 14 for males. For Tgi and Tbody *n* = 15 for females and males

Variable	Unit	Female		Male		Sign	Hedges’ g
		Mean	SD	Mean	SD		
Estimated VO_2_max	mL/kg/min	34.7	9.8	36.1	7.1	0.69	0.16
Metabolism	W	149	24	152	28	0.75	0.11
Metabolism	W/m^2^	90	18	78	18	0.10	− 0.65
Gross efficiency	%	6.9	1.1	6.8	1.3	0.86	− 0.08
WBSR	mg/cm^2^/min	0.25	0.03	0.34	0.10	**0.001**	1.17
LSR	mg/cm^2^/min	0.29	0.11	0.41	0.14	**0.02**	0.92
HR-rest	bpm	69	9	66	12	0.52	− 0.27
HR60	bpm	97	20	94	17	0.64	− 0.16
Tsk-rest	°C	33.7	0.5	34.0	0.7	0.29	0.48
Tsk60	°C	36.4	0.4	36.2	0.3	0.17	− 0.55
Tsk increase	°C/min	0.044	0.007	0.038	0.011	0.07	− 0.63
Tgi-rest	°C	37.0	0.30	36.8	0.24	**0.03**	− 0.72
Tgi60	°C	37.7	0.5	37.5	0.3	0.06	− 0.47
Tgi increase	°C/min	0.012	0.004	0.011	0.005	0.64	− 0.21
Tbody-rest	°C	36.4	0.3	36.3	0.2	0.16	− 0.38
Tbody60	°C	37.5	0.4	37.2	0.3	**0.03**	− 0.83
Tbody increase	°C/min	0.018	0.004	0.016	0.003	0.16	− 0.55
TS 0–60	Dimensionless	1.70	0.93	1.51	0.99	**0.01** #	− 0.19
TC 0–60	Dimensionless	0.54	0.79	0.55	0.64	0.40 #	0.01

## Discussion

To elucidate if elderly females or males are more vulnerable to heat stress, 15 females and 16 males performed low-intensity exercise in a high temperature setting with increasing humidities. The WBGT value corresponding to the temperature/humidity in the first 30 min was 28.9 °C which corresponds to a water vapour pressure of 1.7 kPa or 13 mmHg. Following the 30 min mark, a consistent increase in core temperature (Tgi) was observed, indicating uncompensable heat strain in the elderly participants. The increases in core, skin and mean body temperature during the period of increasing heat stress did not differ between sexes, thereby confirming the hypothesis.

As humidity levels increased, so did the observed difference in evaporated sweat between sexes, in line with our hypothesis. Local evaporative sweat rate was about 22% higher in males than females after 60 min of heat exposure coinciding with a relative humidity nearing 40% (Fig. 2). Despite the lower evaporative sweat rate in females under relatively humid conditions, they managed to constrain the rise in their mean body temperature to values similar to those in males. Excessive sweating beyond the necessary level implies rapid dehydration, potentially leading to an increase in core temperature due to the reduction in circulating blood volume (Sawka et al. 1985). The key challenge is to sweat an amount sufficient for cooling purposes, and females appear to be more adept at achieving this balance. Body core temperatures exceeding values of 42 °C are considered life-threatening (Kosaka et al. 2004). Since no discernible differences in core temperature were observed between sexes after 60 min of heat exposure, we cannot assert the existence of health-related disparities between the sexes in response to heat exposure.

A recent study investigated heat strain in elderly also using a humidity ramp protocol, but did not specifically look at sex differences (Wolf et al. 2023). In their research, uncompensable heat strain typically commenced at relative humidities averaging around 35%, a level higher than observed in our study. Participant characteristics like age (71 ± 6 years versus 69 ± 5 years in our study) and BMI (26 ± 5 kg/m^2^ versus 24 ± 3 kg/m^2^) were comparable. In our study the elderly cycled at 10 W resistance while no resistance was imposed in the US study, but the metabolism (84 ± 15 W/m^2^ at 40 °C versus 84 ± 19 W/m^2^ in our study) was comparable. Gross efficiency in our study was less than 7%. This is common at low workloads—most energy goes to the movement itself, not to the bike (Reger et al. 2013). One possibility is that the US subjects may have undergone some level of heat acclimation, whereas in our study, all experiments were conducted during the winter months.

The amount of evaporated sweat was measured both for the entire body (WBSR) and locally (LSR). These two parameters were highly correlated and of the same magnitude, which suggests that the evaporated sweat measured at the lower arm can be considered as indicative for total evaporated sweat. In dry conditions, sweat production equals sweat evaporation, but in humid circumstances sweat production exceeds sweat evaporation. Females have been shown to produce less sweat than males during heavy exercise Gagnon and Kenny (2012). The observed sex differences in sweat production are likely not related to differences in the number of sweat glands per cm^2^ between sexes (Schwiening et al. 2011) but to activation: females have a lower slope of the sweating response than males (Andérson et al. 1995). Although not significantly different (*p* = 0.07), the increase in Tsk tended to be higher in females than in males (0.044 ± 0.007 °C/min vs. 0.038 ± 0.011 °C/min). This may indicate that females partly compensate for the reduced amount of evaporated sweat by reducing dry heat gain to a larger extent than males.

A recent review on sex differences in thermal sensitivity (Greenfield et al. 2023) reported that most studies, but not all, reported greater warm sensitivity in young women than young men. Elderly females reported to feel a little hot to very hot in 28% of the cases, with males reporting this sensation in only 22% of the cases, showing a similar trend in elderly (Farias et al. 2022). In our study, non-parametric testing showed higher thermal sensation ratings in females than males for the first hour of heat exposure but not in thermal comfort (Table 2). Taken together, females tend to feel warmer than males in similar hot conditions but similarly comfortable.

A limitation of this study is that the findings may not be applicable to the general elderly population in the Netherlands since individuals with underlying health conditions were excluded. Additionally, most participants were recruited from sports facilities, potentially leading to a group that was relatively well-trained. Research indicates that the YMCA test tends to overestimate actual *V*O_2_max in the elderly by about 7.5 ml/kg/min (Takeshima et al. 1992), resulting in actual values of approximately 28 ml/kg/min, considered average for males and above average for females (Letnes et al. 2023). In the older population, individuals are generally less physically fit as they tend to become more sedentary with age (Schutzer and Graves 2004). Furthermore, the general older population often experiences more age-related pathologies that can impact thermoregulation (Meade et al. 2020).

It is undisputed that core temperatures exceeding 42 °C are life-threatening (Kosaka et al. 2004), but is likely that heat related mortality is not directly linked to those extreme core temperature values (Kovats and Hajat 2008). Several heat stress indices are, therefore, available to warn people when critical values in thermal stress and/or strain are reached (Ioannou et al. 2022). One of the most common indicators is the WBGT value. The ISO WBGT-standard threshold for working 8 h at low metabolic rate (125–235 W: 150 W in this study) is set at WBGT 29 °C for non-acclimated adults (ISO_7243 2017). Although these values are specific for workers, they are often also applied to the general public. We observed uncompensable heat strain above 29.5 °C WBGT within a 1 h exposure. Standards are typically established to ensure that only 5% of the population is excluded; however, for the elderly, the average WBGT already approaches the limit values for a fit sample of the aged population. Therefore, it is advisable to consider age-dependent standards to account for this variation.

In conclusion, elderly females exhibit significantly lower sweat rates than their male counterparts during low-intensity exercise at ambient temperatures of 39 °C when humidity exceeds 30%. However, both elderly males and females demonstrate a comparable rise in core temperature, skin temperature, and mean body temperature, indicating similar health-related risks associated with heat exposure.

## Abbreviations

A_D_: Body surface area (m^2^)
BMI: Body mass index (kg/m^2^)
LSR: Local evaporative sweat rate (mg/cm^2^/min)
M: Metabolism (W or W/m^2^)
RH: Relative humidity (%)
Tamb: Ambient temperature (°C)
Tb: Mean body temperature (°C)
TC: Thermal comfort
Tgi: Gastro-intestinal temperature (°C)
TS: Thermal sensation
Tsk: Mean skin temperature (°C)
PSU: Penn state university
RER: Respiratory exchange ratio
VO_2_-max: Maximal oxygen uptake (mL/kg/min)
WBGT: Wet bulb globe temperature (°C)
WBSR: Whole body evaporative sweat rate (mg/cm^2^/min)
